# Assessing the recovery from prerenal and renal acute kidney injury after treatment with single herbal medicine via activity of the biomarkers HMGB1, NGAL and KIM-1 in kidney proximal tubular cells treated by cisplatin with different doses and exposure times

**DOI:** 10.1186/s12906-017-2055-y

**Published:** 2017-12-19

**Authors:** Sung-Man Oh, Gunhyuk Park, Seung Hoon Lee, Chang-Seob Seo, Hyeun-Kyoo Shin, Dal-Seok Oh

**Affiliations:** The K-herb Research Center, Korea Institute of Oriental Medicine, 1672 Yuseong-daero, Yuseong-gu, Daejeon, 34054 Republic of Korea

**Keywords:** Acute kidney injury, Herbal medicine, High-mobility group box protein 1, Neutrophil gelatinase-associated lipocalin, Kidney injury molecule-1

## Abstract

**Background:**

Acute kidney injury (AKI) is an initial factor in many kidney disorders. Pre- and intra-renal AKI biomarkers have recently been reported. Recovery from AKI by herbal medicine has rarely been reported. Thus, this study aimed to investigate the dose- and time-dependent effects of herbal medicines to protect against AKI in cisplatin-induced human kidney 2 (HK-2) cells by assessing the activities of high-mobility group box protein 1 (HMGB1), neutrophil gelatinase-associated lipocalin (NGAL) and kidney injury molecule-1 (KIM-1).

**Methods:**

Proximal tubular HK-2 cell lines were treated with either 400 μM of cisplatin for 6 h or 10 μM of cisplatin for 24 h and then exposed to ten types of single herbal medicines, including *Nelumbo nymphaea* (NY) at a dose of 100 μg/mL. The AKI biomarkers HMGB1, NGAL and KIM-1 were repeatedly measured by an ELISA assay at 2, 4, and 6 h in the group treated with 400 μM of cisplatin to confirm necrotic cell death and at 6, 24, and 48 h in the group treated with 10 μM of cisplatin to examine apoptotic cell death. Recovery confirm was conducted through in vivo study using ICR mice for 3 day NY or *Paeonia* *suffruticosa* intake.

**Results:**

Cisplatin treatment at a concentration of 10 μM decreased cell viability. Treatment with 400 μM of cisplatin reduced HMBG1 activity and resulted in lactate dehydrogenase release. In longer exposure durations (up to 48 h), NGAL and KIM-1 exhibited activity from 24 h onward. Additionally, NY treatment resulted in an approximately 50% change in all three biomarkers. The time-dependent profiles of HMGB1, NGAL and KIM-1 activities up to 48 h were notably different; HMGB1 exhibited a 7-fold change at 6 h, and NGAL and KIM-1 exhibited 1.7-fold changes at 24 h, respectively. Consistently, serum and urine NGAL and KIM-1 activities were all reduced in ICR mice.

**Conclusions:**

Several single herbal medicines, including NY, have a potential as effectors of AKI due to their ability to inhibit the activation of HMGB1, NGAL and KIM-1 in an in vitro AKI-mimicked condition and simple in vivo confirm. Furthermore, an in vivo proof-of-concept study is needed.

**Electronic supplementary material:**

The online version of this article (doi:10.1186/s12906-017-2055-y) contains supplementary material, which is available to authorized users.

## Background

Acute kidney injury (AKI, previously termed acute renal failure) refers to a clinical symptoms characterized by intrinsic renal malfunction [1]. AKI is defined to increase in serum creatinine within 2 to 7 days and the decreased kidney function [2]. Among the AKIs, prerenal and intrinsic AKI frequently coexist that urinary neutrophil gelatinase-associated lipocalin (NGAL), interleukin-18 (IL-18), and albumin show clinically significant elevated concentration in renal disease patients than non-AKI patients do [3]. One of the interesting properties in prerenal AKI is that it is recovered in a reversible manner. During that course, some natural anti-inflammatory or anti-oxidant agents have known to exert their functions to accelerate the recovery.

In the past, herbal medicine has not been studied regarding kidney injury due to the intoxicated episodes by several one. Chinese herbals caused the renal interstitial fibrosis as toxicant roles. i.e., *Stephania tetrandra* contains aristolochic acids, which functions were directly involved in tumorigenesis in rats [4]. Recent attention has been paid to herbal medicine for their potency of ameliorating kidney injuries. Several reports dealing with the benefits of herbals that they promote the main kidney functions of reabsorption, filtration and excretion of glomeruli. From the recent change of perspectives on herbals, we were interested in investigating herbal medicine’s protective effects on acute kidney disorders including prerenal and renal AKIs.

Cisplatin was used to chemotherapeutic agents, derivative of platinum, to treat solid tumors. It was frequently limited by side effects such as ototoxicity, nephrotoxicity [5]. Cisplatin-induced AKI precedes proximal tubular dysfunction and impairment tubular reabsorption [6]. Cisplatin’s higher concentration and shorter-time exposure has been introduced to be an AKI inducible factor [7]. The human kidney 2 (HK-2) cells were treated with higher concentrations of cisplatin versus the treatment with lower concentration presented different cell death either necrosis or apoptosis, respectively [8]. Recent studies have shown the apoptotic phenotypes induced by cisplatin in tubular cells, but those regarding necrosis has been lacking in tubular cells.

Prerenal and renal AKI biomarkers have been listed that neutrophil-gelatinase-associated lipocalin (NGAL), kidney injury molecule-1 (KIM-1) and high-mobility group box protein-1 (HMGB1) have been studied to indicate kidney injury [9]. In the initiation of kidney injuries, each biomarker, HMGB1 and NGAL, KIM-1 have been reported different expressional timelines, different pre-treatment process, even revealed the different cell deaths; acute tubular necrosis or apoptosis [10, 11]. That is why European Medicines Agency recommends them as experimental use as robust kidney injury biomarkers.

It was hypothesized that according to cisplatin concentration and expose timeline, firstly we can set the necrotic and apoptotic normal renal proximal tubular epithelial cell deaths and those setting could be regulated by food and non-food originated herbal medicines, thus, the purpose of the study was to investigate the effects of those herbal medicines on anti-AKI in cisplatin-induced HK-2 cells.

## Methods

### Cell culture

HK-2 human kidney proximal tubule epithelial cells were cultured in keratinocyte serum-free media supplemented with 50 ng/ml bovine pituitary extract and human recombinant epidermal growth factor at a concentration of 5 ng/ml, according to the American Type Culture Collection (ATCC, URL www.atcc.org), in a humidified incubator at 37 °C in 5% CO_2_. The cells were seeded in 96- and 6-well plates at densities of 1 × 10^4^ and 2 × 10^5^ cells/well, respectively.

### Chemicals and reagents

Phosphate buffered saline, penicillin-streptomycin and fetal bovine serum were purchased from Gibco (MD, USA). N-acetylcysteine (NAC) and dimethylsulfoxide were purchased from Sigma-Aldrich (St. Louis, USA). All other reagents used were of guaranteed or analytical grade.

### Herbal raw material acquisition

Ten herbal medicines used in this experiment was purchased from HMAX (Jecheon, Korea), Kwangmyungdang Medicinal Herbs (Ulsan, Korea), and Omniherb (Yeongcheon, Korea) as shown in Additional file 1: Table S1. The origin of the materials was confirmed taxonomically by Prof. Je-Hyun Lee, College of Oriental Medicine, Dongguk University (Gyeongju, Korea) and Prof. Young-Bae Seo, College of Oriental Medicine, Daejeon University (Daejeon, Korea). A voucher specimen of each herbal medicine has been deposited at the K-herb Research Center, Korea Institute of Oriental Medicine (KIOM, Additional file 1: Table S1).

### Preparations of herbal extract

Each dried sample was extracted three times with 70% ethanol by sonication for 60 min or 70% methanol by reflux for 90 min. The extracted solution was filtered through filter paper (No. 2, 150 mm Ø; Whatman, Maidstone, UK) under vacuum, evaporated at 40 °C using BÜCHI R-210 rotary evaporator (Flawil, Switzerland) under vacuum to dryness and then freeze-dried to give a powder using freezing dryer, PVTFD10RS (IlShinBioBase, Yangju, Korea). The amount and yield of extracted samples are summarized in Additional file 2: Table S2.

### Cell viability and kidney injury biomarker detect assays

To evaluate the cell viability, HK-2 cells were seeded onto 96-well plates and then treated with cisplatin in serum-free media for 24 h. The cell viabilities were determined using the Ez-cytox assay kit (DOGEN, Seoul, Korea). After removing the culture media, the cells were stained with a WST assay reagent for 1 h at 37 °C in a 5% CO_2_ incubator. After seeding of HK-2 cells into 6-well plates, the cell were treated with cisplatin, cisplatin + ten herbal medicines for 24 h. Cell culture supernatants were applied into NGAL, KIM-1 pre-coated plates and according to the manufacturer’s instructions (Cloud-Clone Corp, USA, cat. SEB388Hu, SEA785Hu, respectively). To determine whether low and high doses of cisplatin induce apoptosis or necrosis, cells treated with 400 μM of cisplatin were assessed at 2, 4, and 6 h, and cells treated with 10 μM of cisplatin were assessed at 6, 24, and 48 h. The absorbance was determined in lysates at 450 nm using an ELISA reader (VERSA Max; Molecular Devices, Sunnyvale, CA, USA).

### Release of lactate dehydrogenase and extracellular HMGB1 assay

HK-2 cells were seeded in 96 well plate (1 × 10^4^ cells/well) overnight and treated with cisplatin, cisplatin + ten herbal medicines for 6 h. LDH enzyme activity in culture medium was determined by the Cytoscan™ LDH Cytotoxicity Assay Kit according to the manufacture instruction (G-Biosciences, USA, cat. 786-324). HMGB1 release was detected in the culture medium using a sandwich enzyme-linked immune sorbent assay (ELISA) according to the manufacturer’s instructions, and the measurements were performed at 450 nm (Cloud-Clone Corp, USA, cat. SEA399Hu).

### ROS, JC-1 monomers detection, caspase activation and Annexin V binding PI staining

HK-2 cells were seeded in 96 well plate (1 × 10^4^ cells/well) overnight and treated with cisplatin, cisplatin + *Nelumbo* *nymphaea* (NY), *Paeonia* *suffruticosa* (PS) for 30 min. ROS production assays was stained by DCFDA cellular ROS detection assay kit according to the manufacture instruction (Abcam, Cambridge, USA, ab113851). The mitochondrial transmembrane potential was determined with JC-1 dye staining according to the manufacture protocol (Abcam, Cambridge, USA, ab113850). The fluorescence of the JC-1 monomer (green) was read in fluorescence plate reader with excitation/emission setting at 485/535 (Spectramax i3, Molecular Devices. Sunnyvale, USA). Activity of caspases were determined using a colorimetric caspase-9, -3 assay kit (Abcam, Cambridge, USA, ab65608, ab39401, respectively). The assays were performed 60 pi-well plates (5 × 10^5^ cells/well) by incubating cell lysis buffer. After lysis, we measured caspase assay kits according to the manufacture protocol. Apoptotic cell measures were conducted via Annexin V-FITC/PI staining. Adherent cells underwent the collection from centrifugation and then re-suspended in 500 μl 1 × binding buffer. The cells were stained with 5 μL Annexin V–FITC and 5 μl propidium iodide (PI) (50 μg/mL) and incubated at room temperature for 15 min in the dark. The cells were analyzed by a flow cytometry (Becton Dickinson FACS Vantage SE, Sanjose, USA).

### Experimental design

The cells were divided to three group: control, cisplatin-induced group and cisplatin + herbal medicine (dose at 100 μg/mL). Herbal medicines were dissolved in distilled water (D.W.). Herbal medicines were stored at −70 °C until used.

### In vivo study

ICR mice (male, 7 weeks old) were purchased from Daehan Bio Link (ChungBuk, Korea) and acclimated for 1 week before the initiation of experiments. All animal procedures were approved by the Institutional Animal Care and Use Committee of the Korea Institute of Oriental Medicine (16-105). Mice were given an intraperitoneal injection (i.p.) of cisplatin (20 mg/kg) for 3 days. After NY, PS given an oral administration (300 mg/kg) for 3 days. Serum and urine were collected in stored at −20 °C before use.

### Statistical analysis

All experimental results were calculated using *t*-test. All variables were analyzed using the GraphPad Prism 5.10 software (GraphPad Software Inc., San Diego, USA). Each experiment was performed at least three times. Statistical significance was indicated by *p < 0.05, **p < 0.01 and ***p < 0.001.

## Result

### Cisplatin decreases cell viability and induces kidney injury biomarker in HK-2 cells

**Fig. 1:**
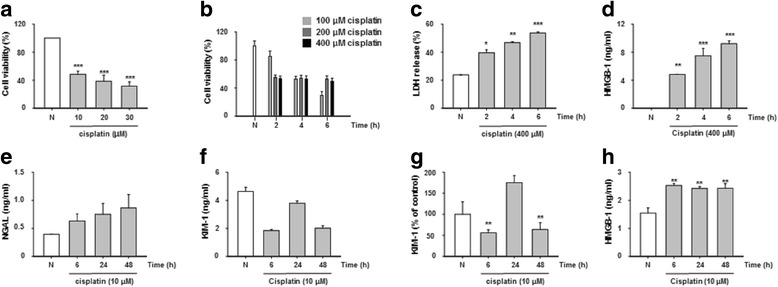
Cisplatin was induced up to cell death and kidney injury biomarkers in HK2 cells. Cell viability of HK-2 cells was treated with cisplatin low dose-long time manner for 24 h (**a**), and high dose-short time treatments for 2 to 6 h (**b**). Release of LDH (**c**) and HMGB1 (**d**) were treated with cisplatin (400 μM) for 2 to 6 h. Secretion levels of NGAL (**e**), KIM-1 (**f**, **g**) and HMGB1 (**h**) for 6 to 48 h. The statistical significance (*p < 0.05, **p < 0.01 and ***p < 0.001) of the observed differences between cisplatin treated and control groups were tested by *t*-test

To assess the toxicity in HK-2 cells, MTT assay was conducted with cisplatin. Treatment with cisplatin for 24 h was decreased viability of HK-2 cells in dose-dependent manner (Fig. 1a). We found that significant decrease in cell viability at high dose and time-dependent manner (Fig. 1b). Because necrosis is characterized by an early disruption of the plasma membrane and cells passively release HMGB1 [12], a high dose of cisplatin (400 μM) resulted in the time-dependent release of HMGB1 and the cellular necrosis indicator LDH in HK-2 cells (Fig. 1c and d). Next, we examined the effect of cisplatin on activity of the biomarkers: NGAL, KIM-1 and HMGB1, which are kidney injury molecules. The activity levels of NGAL and KIM-1 increased by 50% (Fig. 1e–g), which, compared to the control, increased the amount of HMGB1 in the culture media by 50% (Fig. 1h). NGAL, KIM-1 and HMGB1 concentrations can be increased by up to 50% during cisplatin treatment of HK-2 cells.

### Herbal medicines cell viability and their effects on NGAL and KIM-1 molecules

**Fig. 2:**
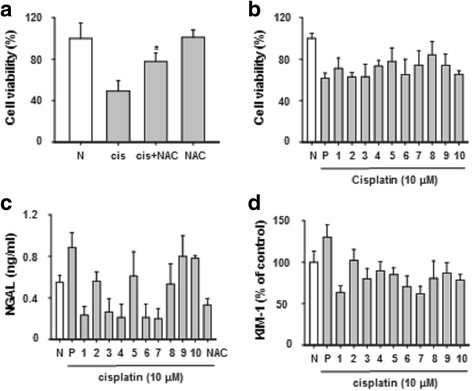
Herbal medicines regulated NGAL and KIM-1 biomarkers. Pretreatment with NAC 10 mM for 1 h and stimulated with cisplatin (10 μM) for 24 h (**a**). Cytotoxicity was measured by MTT assay treatment with herbal medicine and cisplatin for 24 h (**b**). NGAL and KIM-1 were measured by ELISA (**c** and **d**). Lane 1:; *Artemisia capillaris*, Lane 2:; *Houttuynia cordata*, Lane 3:; *Leonurus japonicas*, Lane 4:; *Nelumbo nymphaea*, Lane 5:; *Schisandra chinensis*, Lane 6:; *Akebia quinata*, Lane 7:; *Ligustrum japonicus*, Lane 8:; *Paeonia suffruticosa*, Lane 9:; *Phellodendron amurense*, Lane 10:; *Trichosanthes* *kirilowii*. The statistical significance (*p < 0.05) of the observed differences between cisplatin treated and cisplatin + NAC groups were statistically tested by *t*-test

Initially, we conducted herbal medicine toxicity assay in HK-2 cells that it provided the data on fixing the optimal dose as 100 μg/mL (Additional file 3: Figure S1). To determine whether herbal medicines protect against kidney injuries, we examined cell viability and biomarker expression after herbal medicine treatment in cells exposed to cisplatin. In previous studies, the reactive oxygen species (ROS) scavenger NAC was shown to protect against AKI [13]. An MTT assay was performed in HK-2 cells to investigate the effect of NAC under conditions of cisplatin treatment (Fig. 2a). Compared to the cell viability of cisplatin-treated cells (49%), the cell viability (77%) of cells pretreated with NAC was significantly increased. Additionally, the cell viability assay showed that 10 types of herbal medications protected against cell death (Fig. 2b). Furthermore, levels of the kidney injury biomarkers NGAL and KIM-1 were also markedly decreased from 20 to 50% in cells treated with the 10 types of herbal medicines (Fig. 2c and d).

### HMGB1 was regulated by herbal medicine in high-dose cisplatin-treated HK-2 cells

**Fig. 3:**
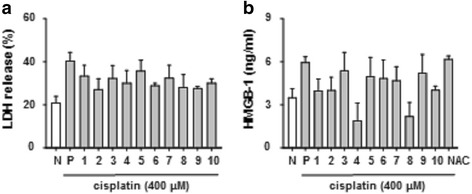
HMGB1 was regulated by herbal medicines with high dose-treated cisplatin in HK-2 cells. HK-2 cells were treated with high – dose of cisplatin (400 μM) for 6 h. Herbal medicines decreased LDH (**a**), and HMGB1 (**b**). The levels of LDH, HMGB1 were measured by ELISA assay kits

During necrotic cell death, HMGB1 is released into the extracellular area and plays important roles as a damage-associated molecular pattern molecule in tubular epithelial cells [14]. Additionally, HMGB1 promotes kidney injury and reduces survival of tubular cells due to ischemia/reperfusion injury [15]. It is unclear whether herbal medicine can block the release of HMGB1 from the cell to the extracellular matrix; we observed that necrotic changes were inhibited by approximately 15% by LDH release (Fig. 3a), and HMGB1 (20 to 50%) was decreased in herbal medicine-treated cells (Fig. 3b). These data indicate that herbal medicines, such as NY and PS, are potent effectors that protect against renal proximal tubular necrotic cell death.

### Profiling of kidney injury biomarker changes that result in necrotic or apoptotic cell death

**Fig. 4:**
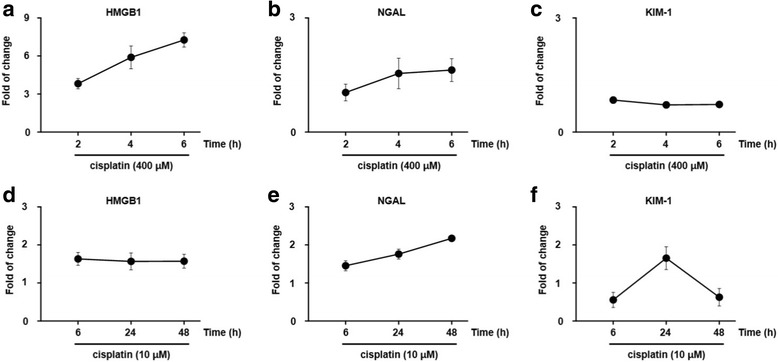
Fold-change of kidney injury biomarkers after high- and low-dose cisplatin treatment. HMGB1 (**a**), NGAL (**b**) and KIM-1 (**c**) levels are shown for cells treated with 400 μM cisplatin for a short exposure time (2 to 6 h). HMGB (**d**), NGAL (**e**) and KIM-1 (**f**) levels were measured in cells treated with 10 μM cisplatin and a longer exposure time (6 to 48 h). The data were compared with the control condition

HMGB1 showed a 4-fold change (2 h), 6-fold change (4 h) and 7-fold change (6 h) in cells treated with 400 μM cisplatin and a 1.6-fold change (6 h), 1.5-fold change (24 h), and 1.5-fold change (48 h) in cells treated with 10 μM cisplatin. NGAL and KIM-1 showed around 1.0-fold change (2 to 6 h) in cells treated with 400 μM cisplatin. NGAL exhibited a 1.4-fold, a 1.7-fold and a 2.1-fold change (6, 24 and 48 h, respectively) in cells treated with 10 μM cisplatin. KIM-1 revealed around 1-fold, a 1.7-fold and 1-fold change (6, 24 and 48 h, respectively) in cells treated with 10 μM cisplatin. (Fig. 4). The data indicated that the expression levels of HMGB1, NGAL and KIM-1 notably varied with dose and time of exposure to cisplatin.

### Cisplatin-induced AKI was related to caspase activation in HK-2 cells death

**Fig. 5:**
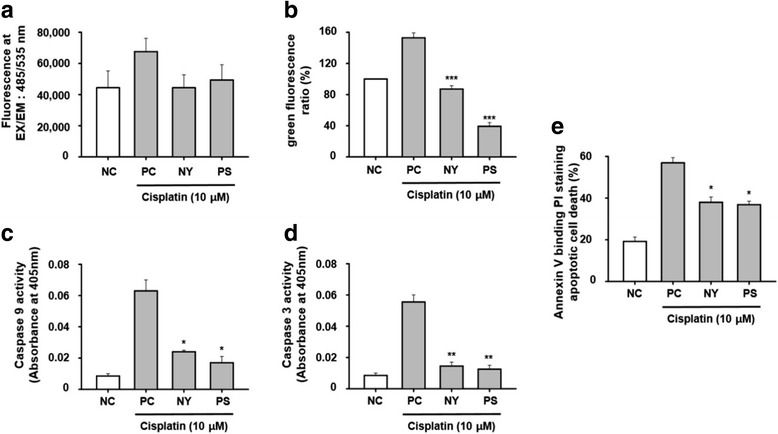
Antioxidant effect of NY and PS on cisplatin-induced cell death in HK-2 cells. Cells were treated with 10 μM cisplatin for 30 m and the level of ROS was detected by microplate reader (**a**). Cells were exposed to cisplatin for 24 h with or without PS or NY (**b**). The measures of JC-1 fluorescence intensity (**c** and **d**). Cells were treated to bind Annexin V and then to undergo PI staining; exposed to cisplatin for 24 h with or without PS or NY. HK-2 cells were treated with cisplatin for 24 h to measure apoptosis (**e**). Cell lysates were assayed for caspase-9, 3 activity. The statistical significance (*p < 0.05, **p < 0.01 and ***p < 0.001) of the observed differences between the cisplatin-treated and herbal groups was determined by a t-test

Cisplatin produces ROS which lead to activation of mitochondrial pathway of apoptosis [16]. We further studied that whether NY, PS inhibits production of ROS and apoptotic molecules. An ROS production and JC-1 green fluorescence were examined in HK-2 cells. NY, PS were decreased to ROS, JC-1 green fluorescence after cisplatin treatments (Fig. 5a and b). Next, further investigated the effect of NY, PS was induced by cisplatin-induced apoptosis, such as caspase-9, -3 activation. The level of caspase-9, -3 activation significantly decreased by NY, PS after cisplatin treatment (Fig. 5c and d). Apoptotic cell measures via Annexin V-FITC/PI staining were statistically different from NY and PS group (Fig. 5e).

### Effects of NY, PS on kidney injury biomarkers, NGAL, KIM-1 in cisplatin-induced mice

**Fig. 6:**
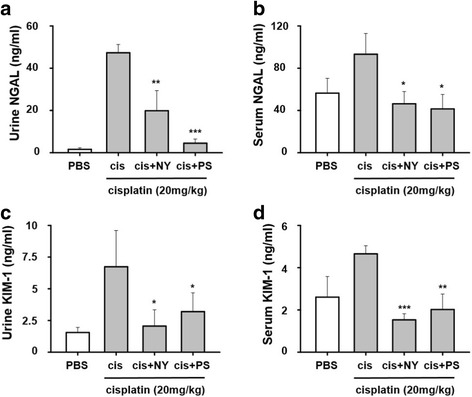
Herbal medicines ameliorated cisplatin-induced kidney injury markers. Mice were given an intraperitoneal injection (i.p.) of cisplatin (20 mg/kg) for 3 days. After *Nelumbo nymphaea* (NY)*, Paeonia suffruticosa* (PS) given an orally administration (300 mg/kg) for 3 days. Concentration of NGAL and KIM-1 were measured in urine and serum (**a**–**d**). All the values are expressed as mean ± S.E.M., *n* = 3 in each group. *, ** and *** statistically different from cisplatin at *p* < 0.05, *p* < 0.01 and *p* < 0.001, respectively

To investigate whether NY, PS, protects kidney injuries, we examined in vivo study. Urinary, serum KIM-1 and NGAL levels a sensitive early time biomarkers cisplatin-induced AKI in mice [17, 18]. Therefore we assessed in mice injected with cisplatin (20 mg/kg) for 3 days and after orally administration *N. nymphaea*, *P. suffruticosa* (300 mg/kg) for 3 days. Urinary and serum NGAL, KIM-1 levels were significantly decreased in cisplatin-induced AKI (Fig. 6a–d). These data implicated that NY, PS had effect on kidney injury biomarkers in cisplatin-induced AKI models.

## Discussion

In this study, herbal medicines ameliorated organic and functional AKI in a dose- and time-dependent manner. NY and PS reduced the elevated levels of NGAL, KIM-1 and HMGB1 activities in ciaplatin-induced in vitro and in vivo settings. The NGAL levels are increased in urine after kidney injury due to the inhibition of kidney reabsorption in the renal tubule [19]. NGAL-induced apoptosis is caused by caspase activation and changes in mitochondrial membrane potential, which in turn increases intracellular iron accumulation [20, 21]. HMGB1 is released by activated tubular cells and macrophages and is passively released by necrotic or damaged cells. Therefore, DAMPs may be secreted and expressed in the cytosol or nucleus and include heat-shock proteins, which cause tissue damage [12, 22]. High-dose cisplatin-induced Poly (ADP-ribose) polymerase 1 activation contributes to necrosis in human, pig and mouse cells [7]. In this study, we observed biomarker modulation by herbal medicines within 6 and 24 h time frames after exposure to cisplatin concentrations of 400 and 10 μM, respectively.

Recently, herbal extracts have been shown to inhibit oxidative stress, inflammation and cisplatin-induced nephrotoxicity. PS can suppress nitric oxide and tumor necrosis factor-α (TNF-α) and is related to the expression of the TLR4-NF-κB pathway in mice [23–25]. NY, *Schisandra chinensis*, and sappanone A are known to reduce cisplatin-induced kidney injury, asthma and chronic obstructive pulmonary disease [26–29]. However, few reports have examined the time- and concentration-dependent effects of NY and PS in cisplatin-induced kidney injuries (Table 1).

**Table 1:** Comparative properties and known information on the studied herbal medicines regarding acute kidney injury

		The studied herbal medicines									
	Herbal Medicine	*Artemisia* *capillaris*	*Houttuynia* *cordata*	*Leonurus* *japonicus*	*Nelumbo* *nymphaea*	*Schisandra* *chinensis*	*Akebia* *quinata*	*Ligustrum* *lucidum*	*Paeonia* *suffruticosa*	*Phellodendron* *amurense*	*Trichosanthes* *kirilowii*
^¶^Assay results in the study	NGAL	++	++	++	+++	++	++	++	++	+	–
	KIM-1	++	+	++	++	++	++	++	++	++	++
	HMGB1	++	++	–	+++	+	+	+	+++	+	+
Information		Liver function recovery activity	Anti-fungal activity and used as a hygiene amenity	Slight anti-uretic activity and used for postpartum hemorrhage	Used for various types of hemorrhage	Increasing urinary flow	Maintains urinary bladder flow	Liver function recovery and anti-uretic effect	Liver function recovery, anti-uretic effect and increasing WBCs	Strong anti-fungal effect; efficacious compound, berberine was developed.	Anti-diabetes and liver dampness effects

The ¶ Mark indicates (−) less than 10% change, a (+) 10–20% change, a (++) 20–50% change, and (+++) greater than 50% change

Induction of kidney injury biomarkers, such as NGAL and KIM-1, and HMGB1, exhibits different profiles during the course of chronological assessment. High-dose and short-term exposure to cisplatin can cause necrotic changes and HMGB1 release. These diagnostic biomarkers may reveal acute tubular necrosis [22]. However, a low dose and a long period after cisplatin treatment resulted in a lower quantity of HMGB1 release. Among the examined herbal medicines, NY and PS decreased HMGB1, NGAL and KIM-1 by 50% compared to the treatment-naïve group. Therefore, further experimental studies are warranted and clinically applicable.

In a previous study, paeonol was isolated from PS and investigated in a mouse model of lipopolysaccharide (LPS)-induced AKI. It also ameliorated serum creatinine and blood urea nitrogen (BUN) levels via modulation of serum inflammatory cytokines, such as TNF-α, IL-1β, and IL-6 [23]. Another of the isolated fractions, protocatechuic aldehyde, was reported to inhibit cisplatin-induced decreases in renal function both in vitro and in vivo by attenuating oxidation and inflammation [30]. In future studies, we could examine the mechanisms by which NY and PS attenuate kidney injury in mice.

Collectively, as shown in Fig. 4 and Table 1, our study provides preliminary but promising evidence on biomarker activity profiles, which depends on the dose of (high or low) and exposure time (short or long) to cisplatin (HMGB1; 7-fold at 6 h with 400 μM; NGAL and KIM-1; 1.7-fold at 24 h with 10 μM). Additionally, further studies should be conducted to prove translational application of herbal medicines for kidney injury patients with abnormal ischemia reperfusion injury conditions or sepsis.

## Conclusions

The present study found that several herbal medicines have beneficial effects on AKI. Additionally, the use of different doses and the time profiles of cisplatin exposure differentially activated the selected kidney injury biomarkers, which increased necrosis and apoptosis in the renal tubular cell line and experimental animal. Ten herbal medicines, including PS and NY, exhibited potential against necrotic and apoptotic cisplatin-induced kidney injuries.

## Additional files

Additional file 1: Table S1. List of herbal medicines. (DOCX 14 kb)

Additional file 2: Table S2. Sample information. (DOCX 12 kb)

Additional file 3: Figure S1. MTT assay was conducted with herbal medicines which dose range from 0.1 to 1 mg/ml was treated to herbal medicines for 24 h. The 0.1 mg/ml concentration was chosen as the fixed dose. The cell viability of HK-2 cells treated with dose dependent herbal medicines. Lane 1: *Artemisia capillaris*, Lane 2: *Houttuynia cordata*, Lane 3: *Leonurus japonicas*, Lane 4: *Nelumbo nymphaea*, Lane 5: *Schisandra chinensis*, Lane 6: *Akebia quinata*, Lane 7: *Ligustrum japonicus*, Lane 8: *Paeonia suffruticosa*, Lane 9: *Phellodendron amurense*, Lane 10: *Trichosanthes kirilowii*. (JPEG 81 kb)

## Abbreviations

AKI: Acute kidney injury
ATCC: American Type Culture Collection
BUN: Blood urea nitrogen
HMGB1: High mobility group box protein 1
IL-18: Interleukin-18
KIM-1: Kidney injury molecule-1
NAC: N-acetylcysteine
NGAL: Neutrophil gelatinase-associated lipocalin
NY: *Nelumbo nymphaea*
PS: *Paeonia suffruticosa*
ROS: Reactive oxygen species
SCr: Serum creatinine
