# Supplementary material for: Assessing the recovery from prerenal and renal acute kidney injury after treatment with single herbal medicine via activity of the biomarkers HMGB1, NGAL and KIM-1 in kidney proximal tubular cells treated by cisplatin with different doses and exposure times
Source: BMC Complement Altern Med. 2017 Dec 19;17:544. doi: 10.1186/s12906-017-2055-y (PMC5738030; doi:10.1186/s12906-017-2055-y)
Supplement: Supplementary file 1 — List of herbal medicines. (DOCX 14 kb) [file 12906_2017_2055_MOESM1_ESM.docx]

**Additional file 1:** **Table S1.** List of herbal medicines.

| Scientific name | Latine name | Family | Used part | Company of purchase | Source |
| --- | --- | --- | --- | --- | --- |
| *Houttuynia cordata* Thunb. | Houttuyniae Herba | Saururaceae | Herba | HMAX | China |
| *Phellodendron amurense* Rupr. | Phellodendri Cortex | Rutaceae | Bark | Kwangmyungdang Medicinal Herbs | China |
| *Artemisia capillaris* Thunb. | Artemisiae Capillaris Herba | Compositae | Herba | Kwangmyungdang Medicinal Herbs | Korea |
| *Paeonia suffruticosa* Andr. | Moutan Radicis Cortex | Paeoniaceae | Root bark | Kwangmyungdang Medicinal Herbs | Korea |
| *Akebia quinata* Dcne. | Akebiae Caulis | Lardizabalaceae | Stem | Kwangmyungdang Medicinal Herbs | Korea |
| *Ligustrum japonicus* Thunb. | Ligustri Fructus | Oleaceae | Fruit | Kwangmyungdang Medicinal Herbs | China |
| *Nelumbo nucifera* Gaertn. | Nelumbinis Semen | Nymphaeaceae | Seed | Kwangmyungdang Medicinal Herbs | China |
| *Leonurus japonicus* Houtt. | Leonuri Herba | Labiatae | Herb | Kwangmyungdang Medicinal Herbs | Korea |
| *Trichosanthes kirilowii* Maxim. | Trichosanthis Radix | Cucurbitaceae | Root | Kwangmyungdang Medicinal Herbs | Korea |
| *Schisandra chinensis* (Turcz.) Baill. | Schisandrae Fructus | Schisandraceae | Fruit | Omniherb | Korea |
