# Supplementary material for: Assessing the recovery from prerenal and renal acute kidney injury after treatment with single herbal medicine via activity of the biomarkers HMGB1, NGAL and KIM-1 in kidney proximal tubular cells treated by cisplatin with different doses and exposure times
Source: BMC Complement Altern Med. 2017 Dec 19;17:544. doi: 10.1186/s12906-017-2055-y (PMC5738030; doi:10.1186/s12906-017-2055-y)
Supplement: Supplementary file 2 — Sample information. (DOCX 12 kb) [file 12906_2017_2055_MOESM2_ESM.docx]

**Additional file 2: Table S2.** Sample information.

| Latine name | Voucher specimen | Extract method | Extract weight (g) | Yield (%) |
| --- | --- | --- | --- | --- |
| *Houttuyniae Herba* | 2008-KIOM17 | Sonication | 12.6 | 6.3 |
| *Phellodendri Cortex* | 2009-KIOM18 | Sonication | 44.4 | 14.8 |
| *Artemisiae Capillaris Herba* | 2009-KIOM30 | Sonication | 24.8 | 8.3 |
| *Moutan Radicis Cortex* | 2009-KIOM42 | Sonication | 146.8 | 20.9 |
| *Akebiae Caulis* | 2009-KIOM43 | Sonication | 72.8 | 10.4 |
| *Ligustri Fructus* | 2009-KIOM51 | Sonication | 111.9 | 15.9 |
| *Nelumbinis Semen* | 2009-KIOM52 | Sonication | 69.7 | 9.9 |
| *Leonuri Herba* | 2009-KIOM56 | Sonication | 91.9 | 13.1 |
| *Trichosanthis Radix* | 2015-KIOM106 | Sonication | 77.2 | 7.7 |
| *Schisandrae Fructus* | 2009-KIOM69 | Reflux | 48.0 | 48.0 |
