# Supplementary figures and images for: Assessing the recovery from prerenal and renal acute kidney injury after treatment with single herbal medicine via activity of the biomarkers HMGB1, NGAL and KIM-1 in kidney proximal tubular cells treated by cisplatin with different doses and exposure times
Source: BMC Complement Altern Med. 2017 Dec 19;17:544. doi: 10.1186/s12906-017-2055-y (PMC5738030; doi:10.1186/s12906-017-2055-y)

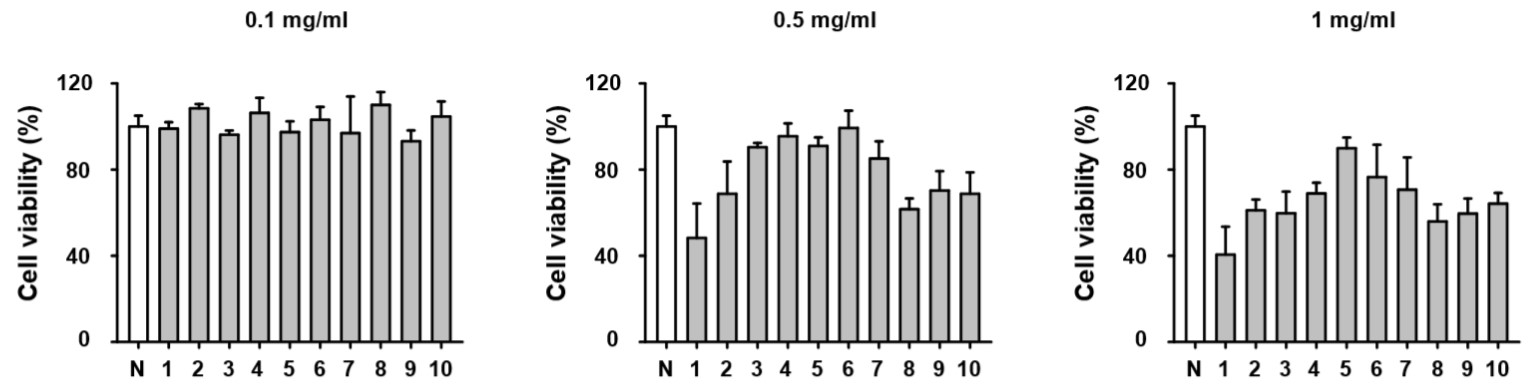

Supplement: Supplementary file 3 — MTT assay was conducted with herbal medicines which dose range from 0.1 to 1 mg/ml was treated to herbal medicines for 24 h. The 0.1 mg/ml concentration was chosen as the fixed dose. The cell viability of HK-2 cells treated with dose dependent herbal medicines. Lane 1: Artemisia capillaris, Lane 2: Houttuynia cordata, Lane 3: Leonurus japonicas, Lane 4: Nelumbo nymphaea, Lane 5: Schisandra chinensis, Lane 6: Akebia quinata, Lane 7: Ligustrum japonicus, Lane 8: Paeonia suffruticosa, Lane 9: Phellodendron amurense, Lane 10: Trichosanthes kirilowii. (JPEG 81 kb) [file 12906_2017_2055_MOESM3_ESM.jpg]
